# A versatile chemical conversion synthesis of Cu_2_S nanotubes and the photovoltaic activities for dye-sensitized solar cell

**DOI:** 10.1186/1556-276X-9-513

**Published:** 2014-09-19

**Authors:** Xuemin Shuai, Wenzhong Shen, Zhaoyang Hou, Sanmin Ke, Chunlong Xu, Cheng Jiang

**Affiliations:** 1 Department of Applied Physics, Chang’an University, Xi’an 710064, China; 2 Laboratory of Condensed Matter Spectroscopy and Opto-Electronic Physics and Key Laboratory of Artificial Structures and Quantum Control (Ministry of Education), Department of Physics, Shanghai Jiao Tong University, Shanghai 200240, China; 3 School of Physics and Electronic Electrical Engineering, Huaiyin Normal University, 111 West Chang Jiang Road, Huaian 223300, China

**Keywords:** Nanotubes, Chemical transformation, Cation exchange, Growth mechanism, Optical and photovoltaic properties

## Abstract

A versatile, low-temperature, and low-cost chemical conversion synthesis has been developed to prepare copper sulfide (Cu_2_S) nanotubes. The successful chemical conversion from ZnS nanotubes to Cu_2_S ones profits by the large difference in solubility between ZnS and Cu_2_S. The morphology, structure, and composition of the yielded products have been examined by field-emission scanning electron microscopy, transmission electron microscopy, and X-ray diffraction measurements. We have further successfully employed the obtained Cu_2_S nanotubes as counter electrodes in dye-sensitized solar cells. The light-to-electricity conversion results show that the Cu_2_S nanostructures exhibit high photovoltaic conversion efficiency due to the increased surface area and the good electrocatalytical activity of Cu_2_S. The present chemical route provides a simple way to synthesize Cu_2_S nanotubes with a high surface area for nanodevice applications.

## Background

Since the discovery of carbon nanotubes in 1991 by Iijima [1], nanotubes have become a symbol of the new and fast-developing research area of nanotechnology due to their significant potential applications in optoelectronics, advanced catalysis, biotechnology, separation, memory devices, and so on [2-8]. A variety of nanotubes, such as metals and semiconductors [5,9], the so-called functional materials, have so far been prepared by various approaches including hydrothermal method, sol-gel technique [10], template-assisted method [11,12], electroless deposition [13], surfactant intercalation method, microwave-enhanced synthesis [14], and thermal evaporation method [15]. At present, template-based techniques turn out to be particularly effective for growth of nanotubes in spite of complicated processes involved [16,17]. However, the template removal process after nanotube formation inevitably affects the purity of the materials and may also cause the partial loss of nanotube orientation [18]. Hence, it is necessary to explore a simple and efficient synthesis method for preparing one-dimensional tubular nanostructures in large quantities without additional surfactants or templates.

Copper sulfide (Cu_2_S), an indirect semiconductor with a bulk bandgap of 1.21 eV [19,20], has extensively been investigated and is widely used in field emission [21], switching [22], sensing devices [23], and solar cells in virtue of its relatively high electrocatalytic activity [24,25]. The availability of Cu_2_S nanostructures with well-defined morphologies and dimensions should enable bringing new types of applications or enhancing the performance of currently existing photoelectric devices due to the quantum size effects. Therefore, the synthesis of Cu_2_S materials with well-controlled size and shape is of great significance for their applications. Until now, a variety of nanostructures of Cu_2_S such as nanowires [26,27], nanoparticles [28], nanodisks [29], nanocrystals [30,31], and nanoplates [32] have already been synthesized by various methods. Nevertheless, little has been devoted to the development of a general and low-cost synthetic method to fabricate Cu_2_S nanotubes without using any templates or crystal seeds. Considering that size and morphology are crucial factors in determining the properties of nanomaterials, the control over them is of great interest with regard to specific applications of such materials as nanodevices.

In this article, we describe a novel route for the synthesis of Cu_2_S nanotubes by conversion from ZnS nanotubes via a chemical conversion and cation exchange process at a low temperature of 90°C. Our previous studies on the transformation of composition have indicated the significance of chemical conversion and cation exchange [33-36]. The basic idea behind this route is to take advantage of the large difference in solubility between ZnS and Cu_2_S for effective transformation. Moreover, we have shown high photovoltaic performances of Cu_2_S nanotubes as the counter electrodes in dye-sensitized solar cells (DSSCs), due to the enormous surface area and good electrocatalytical activity of Cu_2_S [25,37]. The present technique is very convenient and versatile with the advantages of simplicity (free of any special equipment or templates), mild condition (low growth temperature), and high yield (near 100% morphological yield) and has been demonstrated to control and manipulate effectively the chemical compositions and structures of nanotubes.

## Methods

### Synthesis of ZnS nanotubes

The preparation details for ZnS nanotubes can be found in our recently published papers [35,36]. Briefly, ZnO nanowires were first prepared by a hydrothermal process. As a typical synthesis process, 0.2 g ZnCl_2_ and 20.0 g Na_2_CO_3_ were added into a 50-mL Telfon-lined stainless steel autoclave and filled with distilled water up to 90% of its volume. After vigorous stirring for 30 min, the autoclave was maintained at 140°C for 12 h, followed by cooling down naturally to room temperature. The synthesis of ZnO nanowires could be realized after the product was washed and dried. Subsequently, the as-prepared ZnO nanowires on substrates (silicon or glass slides) were transferred to a Pyrex glass bottle containing 40 mL 0.2 M thioacetamide (TAA). The sealed bottle was then heated to 90°C for 9 h in a conventional laboratory oven to synthesize ZnS nanotubes. The final products on the substrates were washed repeatedly with deionized water and then dried at 60°C before being used for the next step in the reaction and further characterization.

### Synthesis of Cu_2_S nanotubes

The synthesis of Cu_2_S nanotubes was realized by transferring the silicon or glass slides with ZnS nanotubes on them to a Pyrex glass bottle containing 20 mM CuCl and 70 mM tartaric acid. During the reaction process, the solution temperature was kept at 90°C. The final products on the substrates were washed thoroughly using deionized water to remove any co-precipitated salts and then dried at air at 60°C. For better crystal quality and stability, the as-prepared Cu_2_S nanotubes were annealed at 200°C for 10 min under argon atmosphere.

### Morphological and structural characterization

The morphology and structure of the samples were characterized using a field-emission scanning electron microscope (FE-SEM; Philips XL30FEG, FEI Co., Hillsboro, OR, USA) with an accelerating voltage of 5 kV and a high-resolution transmission electron microscope (HRTEM; JEOL JEM-2100 F, JEOL Ltd., Akishima, Tokyo, Japan). Selected area electron diffraction (SAED) and energy-dispersive X-ray (EDX) microanalysis were also performed during the transmission electron microscopy (TEM) and scanning electron microscopy (SEM) observations. X-ray diffraction (XRD) was carried out on a diffractometer (D/max-2200/PC, Rigaku Corporation, Tokyo, Japan) equipped with a high-intensity Cu Kα radiation (*λ* = 1.5418 Å). Raman spectra were measured at room temperature on a Jobin Yvon LabRAM HR 800UV micro-Raman/PL system (HORIBA Jobin Yvon Inc., Edison, NJ, USA) at the backscattering configuration under the excitation of a He-Cd laser (325.0 nm) for ZnS nanotubes but Ar^+^ laser (514.5 nm) for Cu_2_S nanotubes.

### Fabrication of DSSCs

The TiO_2_ nanoporous films with an area of 0.25 cm^2^ were sintered in air for 1 h at 500°C and then immersed in 0.5 mM N719 dye (Ruthenium 535-bisTBA, Solaronix, Aubonne, Switzerland) solution in ethanol for 12 h. These films were used as the photoanodes and mounted together with a counter electrode with Cu_2_S nanotubes (prepared by coating on fluorine-doped tin oxide (FTO) glass) to form backside illuminated cells. The Cu_2_S-coated FTO glass was prepared by drop-casting Cu_2_S solution on the clean FTO glass and subsequently waiting until all solvent evaporates. The liquid electrolyte was injected into the cells by a syringe, which consisted of 0.1 M iodine (I_2_), 0.1 M lithium iodide (LiI), 0.6 M tetra-butylammonium iodide, and 0.5 M 4-*tert*-butyl pyridine in acetonitrile (CH_3_CN, 99.9%).

## Results and discussion

**Figure 1:**
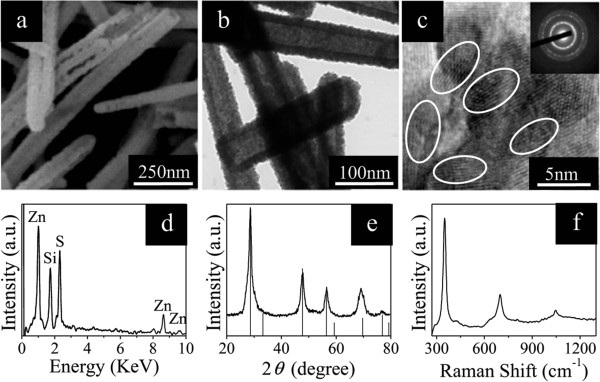
**FE-SEM, TEM, and HRTEM images and EDX, XRD, and Raman spectra of ZnS nanotubes. (a)** FE-SEM and **(b)** TEM images of ZnS nanotubes. **(c)** HRTEM image of a ZnS nanotube shell, together with the corresponding SAED pattern shown in the inset. The corresponding **(d)** EDX, **(e)** XRD, and **(f)** room-temperature Raman spectra of ZnS nanotubes.

In our experiments, ZnO nanowires were first prepared by a hydrothermal process. Conversion to ZnS nanotubes was then obtained by transferring ZnO nanowires into TAA solution. Typically, samples were heated at 90°C for 9 h [35]. We believe that this result may be explained by a fast out-diffusion of Zn ions and a less efficient in-diffusion of S [38]. Figure 1a shows the FE-SEM image of the obtained ZnS nanotubes. The irregular open tips on some of the shells authenticate the hollow nature of the prepared nanotubes. TEM image (Figure 1b) gives further evidence for the hollow structure of ZnS nanotubes. The diameters of ZnO nanowires and ZnS nanotubes are about 70 nm. Figure 1c presents a HRTEM image taken on the edge of the ZnS nanotube, which exhibits clear crystal lattice fringes without noticeable structural defects. The corresponding ringlike SAED pattern (inset of Figure 1c) also provides evidence for the polycrystalline nature of ZnS nanotubes. The composition of the ZnS nanotubes can be easily identified by the EDX spectrum (Figure 1d). Measurements of the XRD pattern (Figure 1e) and the room-temperature Raman spectrum (Figure 1f) also confirm that the reaction product is ZnS. The observation of multiple resonant Raman peaks indicates that the yielded ZnS nanotubes possess good optical quality [39].

**Figure 2:**
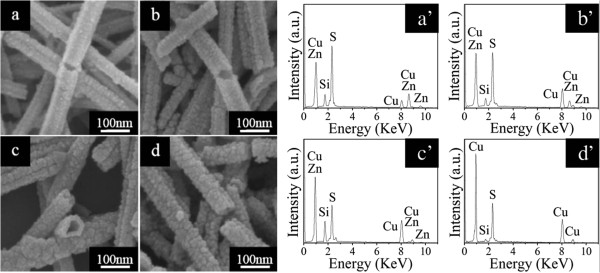
**FE-SEM images and EDX spectra of Cu**_**2**_**S nanotubes with different reaction times.** FE-SEM images of Cu_2_S nanotubes with different reaction times: **(a)** 10 min, **(b)** 20 min, **(c)** 40 min, and **(d)** 1 h. **(a’-d’)** The corresponding EDX spectra of Cu_2_S nanotubes with different reaction times.

The main attempt in the present work is to synthesize Cu_2_S nanotubes and to investigate their optical properties and photovoltaic conversion efficiency when used as a counter electrode. To make the conversion of ZnS nanotubes to Cu_2_S ones, we transfer the substrates with ZnS nanotubes on them into 40 mL of 20 mM CuCl and 70 mM tartaric acid aqueous solution. When immersed into the abovementioned solutions, the ZnS surface turned dark red immediately, and then shinning cyan and gray in a short time. After 1 h’s reaction, the product surface became black and fluffy, manifesting the formation of dense Cu_2_S nanotubes. A series of time-dependent experiments were conducted to track the formation process of Cu_2_S tubular structures, as shown in Figure 2. Under the reaction time of 10 min, some Cu_2_S nanoparticles on the ZnS nanotubes were observed because ion exchange happens as Cu^+^ reacts with S^2-^ slowly dissolved from the surface of ZnS nanotubes to form initial Cu_2_S shells, as depicted in Figure 2a. After another 10 min’s reaction, more Cu_2_S nanoparticles piled up on the initial Cu_2_S shells (Figure 2b). When the reaction time reached to 40 min, large numbers of Cu_2_S nanoparticles were produced (Figure 2c). When further prolonging the reaction time to 1 h, uniform Cu_2_S nanotubes of large quantities with diameters of about 70 nm and lengths of about 300 to 500 nm were fully converted from ZnS ones (Figure 2d).

The corresponding EDX spectra in Figure 2a’,b’,c’,d’ give clear evidence for the FE-SEM observation of the samples obtained through various reaction times. From Figure 2a’, we can observe the successful incorporation of Cu element into the ZnS nanotubes in the compositional information, and the Cu/Zn stoichiometric ratio is 0.47. The signal of Si originates from the substrate. With the increase of the reaction time, the Cu/Zn stoichiometric ratio becomes higher and higher (from 1.21 to 2.82) due to the fact that more and more Zn atoms were replaced by Cu atoms with the reaction processing, as shown in Figure 2b’,c’. Further chemical reaction will yield pure Cu_2_S nanotubes, which can be unambiguously confirmed by the EDX spectrum in Figure 2d’. There are only Cu, S, and Si elements without any Zn element, and the Cu/S stoichiometric ratio is 2.0. This result confirms the total exchange of cations during the transformation process from ZnS to Cu_2_S.

According to the experimental observation described above, the whole process can be described as follows. Once the obtained ZnS nanotubes were transferred into CuCl solution, cation exchange began at the interfaces between the ZnS nanotube surfaces and solution. With the increase in the reaction time, Zn^2+^ was gradually substituted by Cu^+^, resulting in the synthesis of Cu_2_S nanotubes. The driving force for the cation exchange is provided by the large difference in solubility between ZnS and Cu_2_S (solubility product constant (*K*_sp_) of ZnS is 2.93 × 10^-25^, whereas *K*_sp_ of Cu_2_S is 2.5 × 10^-48^) [40]. The above conversion mechanism reveals that the ZnS nanotubes can act as both reactants and templates during the cation-exchange process.

**Figure 3:**
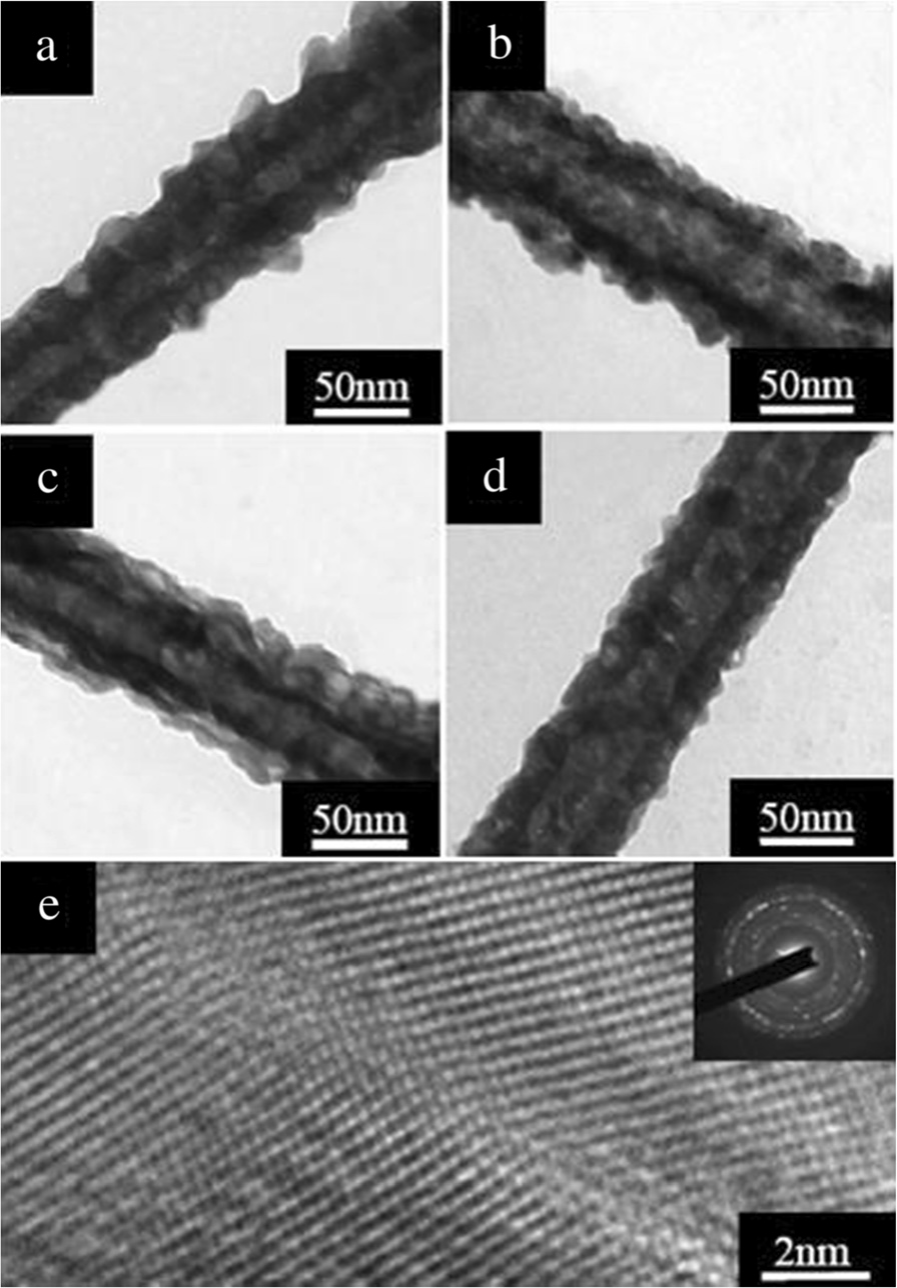
**TEM and HRTEM images and SAED pattern of the Cu**_**2**_**S nanotubes.** TEM images of the Cu_2_S nanotubes with different reaction times: **(a)** 10 min, **(b)** 20 min, **(c)** 40 min, and **(d)** 1 h. **(e)** HRTEM image of the Cu_2_S-1 h nanotubes, together with the corresponding SAED pattern shown in the inset.

Samples were analyzed by TEM to determine the morphology of the cation-exchanged products. Figure 3a shows the TEM image of the as-prepared Cu_2_S nanotubes obtained at 10 min. One can notice that bits of Cu_2_S nanoparticles with an average size of 18 nm were formed on the outer layers of ZnS nanotubes. As the reaction time reached 20 min, the Cu_2_S nanoparticles on the surface of nanotubes became a bit more, as seen in Figure 3b. With the reaction time increased to 40 min, the TEM in Figure 3c reveals that the outer layers were composed of numerous Cu_2_S nanoparticles. Further prolonging the chemical reaction time to 1 h, we were able to realize uniform and pure Cu_2_S nanotubes with about 70 nm in diameter and 18 to 22 nm in shell thickness (Figure 3d).

HRTEM analyses were performed on Cu_2_S-1 h nanotubes to obtain detailed information regarding the structure of the nanotubes. Figure 3e is a representative HRTEM image taken on the edge of the obtained Cu_2_S-1 h nanotube (Figure 3d). Only the polycrystalline nature of Cu_2_S nanotubes can be observed. The clearly observed crystal lattice fringes demonstrate that the nanotubes are highly crystallized and free from dislocation and stacking faults. The corresponding SAED pattern with characteristic ring diffractions shown in the inset of Figure 3e also confirms the polycrystalline feature of the Cu_2_S nanotubes.

**Figure 4:**
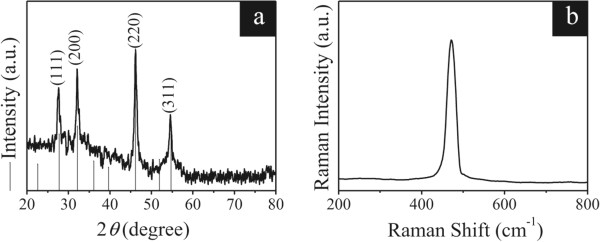
**XRD pattern (a) and room-temperature Raman spectrum (b) of the Cu**_**2**_**S-1 h nanotubes.**

The XRD pattern of the samples prepared by chemical conversion and cation exchange is shown in Figure 4a. The diffraction peaks of Cu_2_S-1 h nanotubes can be indexed to a single phase of cubic Cu_2_S (JCPDS File No. 53-0522). The shape of the diffraction peaks demonstrates that the products should be well crystallized. No other impurities were found in the samples, indicating that the products are pure cubic Cu_2_S.

Raman spectroscopy is an effective tool for the study of the molecular structure within nanostructures. Up to now, there is little research work on the Raman characterization of Cu_2_S nanostructures. Figure 4b shows the room-temperature Raman spectrum of the Cu_2_S-1 h nanotubes. The excitation wavelength is 514.5 nm from an Ar ion laser. A strong and sharp band at 472 cm^-1^ probably originates from the lattice vibration, which is consistent with the results reported for Cu_2_S films [41,42] and Cu_2_S nanotree arrays [43].

**Figure 5:**
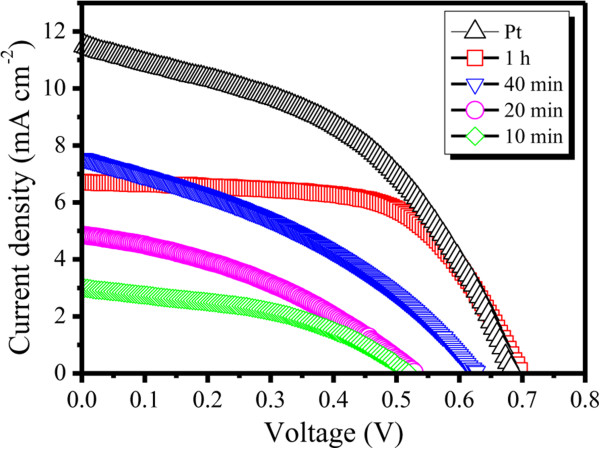
**Photovoltaic behavior of dye-sensitized solar cells with counter electrodes of Cu**_**2**_**S nanotubes at different reaction times.** Under illumination of 100 mW cm^-2^.

To characterize the influence of Cu_2_S on the performance of counter electrodes, a series of time-dependent *J*-*V* curves are shown in Figure 5 and the photovoltaic parameters of the tested DSSCs are listed in Table 1. When the Cu_2_S nanotubes processed by various reaction times were applied into DSSCs, the cell performance was increased significantly as the reaction time increases from 10 min to 1 h. Both the photocurrent and photovoltage increased with reaction time, and they reached the peak value when the reaction time reached 1 h. The improved efficiency can be attributed to the larger specific surface area of the produced Cu_2_S nanoparticles since the enlarged surface helps to increase the photovoltaic reaction sites and promote the efficiency of the electron-hole separation [37], and the composition of the nanotubes gradually changing from ZnS through mixed ZnCuS to Cu_2_S. Furthermore, the best photovoltaic conversion efficiency (*η*) up to 2.88% was achieved at 1 h’s reaction time with the parameters of 6.715 mA cm^-2^ in short-circuit current density (*J*_sc_), 0.70 V in open-circuit voltage (*V*_oc_), and 0.62 in fill factor (FF), which indicates the high electrocatalytical activity of Cu_2_S reported by Hodes et al. [25]. Therefore, the large surface area of the Cu_2_S nanotubes was not the only factor responsible for the high photovoltaic performance, and the good electrocatalytical activity could also be critical.

**Table 1:** **Photovoltaic parameters of tested DSSCs using Pt and Cu**_**2**_**S nanotubes of different reaction times as counter electrodes**

	***J***_**sc **_**(mA cm**^**-2**^**)**	***V***_**oc **_**(V)**	**FF**	***η *** **(%)**
Cu_2_S-10 min	2.99	0.52	0.43	0.67
Cu_2_S-20 min	4.86	0.53	0.37	0.95
Cu_2_S-40 min	7.55	0.64	0.36	1.72
Cu_2_S-1 h	6.72	0.70	0.62	2.88
Pt	11.50	0.68	0.44	3.50

For comparison, the photovoltaic performance of DSSC with Pt counter electrode is shown in Figure 5 and the photovoltaic parameters are listed in Table 1 while keeping other factors unchanged. Although the performances of Cu_2_S counter electrode DSSCs are slightly inefficient in photovoltaic conversion efficiency (*η*), it is noteworthy that the cost reduction is crucial for future development all the time for all kinds of solar cells, which means our Cu_2_S counter electrodes are completely competent for application in high-efficiency dye-sensitized solar cells.

## Conclusions

In summary, we have developed a versatile chemical conversion synthesis of Cu_2_S nanotubes at a low temperature of 90°C. The conversion mechanism of the Cu_2_S nanotubes from ZnS nanotubes is due to the large difference in solubility between ZnS and Cu_2_S. The morphological, structural, and optical characteristics of the yielded Cu_2_S nanotubes were characterized by SEM, TEM, XRD, and Raman spectra in detail. Furthermore, the prepared Cu_2_S nanostructures have been successfully used as the counter electrodes in dye-sensitized solar cells. Compared to all those Cu_2_S nanotubes produced at different reaction times, the photovoltaic efficiency was enhanced significantly as the reaction time increases from 10 min to 1 h, and also a photovoltaic conversion efficiency up to 2.88% was obtained. We attribute the improved performance to the increased surface area and the good electrocatalytical activity of Cu_2_S. An optimized process to prepare the Cu_2_S DSSCs is expected to further promote the overall efficiency. Although the current work focuses on the synthesis and application of Cu_2_S nanotubes in dye-sensitized solar cells, this kind of nanostructures is also expected to be used in other nanodevices such as gas sensors, photocatalyzers, quantum dot-sensitized solar cells, and so on, in which a high surface area is preferred. The present strategy is a very convenient and efficient method to control and manipulate effectively the chemical composition and structure of nanomaterials. This simple chemical method opens up possibilities to the synthesis of various nanostructures with high surface area for extensive study of the physical and chemical properties of the obtained nanostructures, broadening their potential nanodevice applications.

## Abbreviations

DSSCs: dye-sensitized solar cells; EDX: energy-dispersive X-ray; FE-SEM: field-emission scanning electron microscopy; HRTEM: high-resolution transmission electron microscopy; SAED: selected area electron diffraction; TAA: thioacetamide; XRD: X-ray diffraction.

## Competing interests

The authors declare that they have no competing interests.

## Authors’ contributions

XMS participated in the design of the study, carried out the experiments, and performed the statistical analysis as well as drafted the manuscript. WZS took charge of the design of the study, provided the theoretical and experimental guidance, and revised the manuscript. SMK, ZYH, CJ, CLX participated in the design and coordination of the study and helped to draft the manuscript, and CJ contributed a lot to the revisions of the manuscript. All authors read and approved the final manuscript.

## References

[B1] Iijima S Helical microtubules of graphite carbon Nature 1991 354 56 58 10.1038/354056a0

[B2] Xia YN Yang PD Sun YG Wu YY Mayers B Gates B Yin YD Kim F Yan HQ One-dimensional nanostructures: synthesis, characterization, and applications Adv Mater 2003 15 353 389 10.1002/adma.200390087

[B3] Haradam M Adachi M Surfactant-mediated fabrication of silica nanotubes Adv Mater 2000 12 839 841 10.1002/(SICI)1521-4095(200006)12:11<839::AID-ADMA839>3.0.CO;2-9

[B4] Hu JT Odom TW Lieber CM Chemistry and physics in one dimension: synthesis and properties of nanowires and nanotubes Acc Chem Res 1999 32 435 445 10.1021/ar9700365

[B5] Xiong YJ Mayers BT Xia YN Some recent developments in the chemical synthesis of inorganic nanotubes Chem Commun 2005 5013 5022 10.1039/b509946c 16220157

[B6] Remskar M Inorganic nanotubes Adv Mater 2004 16 1497 1504 10.1002/adma.200306428

[B7] Martin CR Kohli P The emerging field of nanotube biotechnology Nat Rev Drug Discov 2003 2 29 37 10.1038/nrd988 12509757

[B8] Lee SB Mitchell DT Trofin L Nevanen TK Soderlund H Martin CR Antibody-based bio-nanotube membranes for enantiomeric drug separations Science 2002 296 2198 2200 10.1126/science.1071396 12077410

[B9] Goldberger J Fan R Yang PD Inorganic nanotubes: a novel platform for nanofluidics Acc Chem Res 2006 39 239 248 10.1021/ar040274h 16618091

[B10] Kovtyukhova NI Mallouk TE Mayer TS Templated surface sol–gel synthesis of SiO_2_ nanotubes and SiO_2_-insulated metal nanowires Adv Mater 2003 15 780 785 10.1002/adma.200304701

[B11] Niu HJ Gao MY Diameter-tunable CdTe nanotubes templated by 1D nanowires of cadmium thiolate polymer Angew Chem Int Ed 2006 45 6462 6466 10.1002/anie.200601779 16897792

[B12] Fan R Wu YY Li DY Yue M Majumdar A Yang PD Fabrication of silica nanotube arrays from vertical silicon nanowire templates J Am Chem Soc 2003 125 5254 5255 10.1021/ja034163+ 12720419

[B13] Yan CL Xue DF Electroless deposition of aligned ZnO taper-tubes in a strong acidic medium Electrochem Commun 2007 9 1247 1251 10.1016/j.elecom.2007.01.029

[B14] Cao XB Zhao C Lan XM Gao GJ Qian WH Guo Y Microwave-enhanced synthesis of Cu_3_Se_2_ nanoplates and assembly of photovoltaic CdTe-Cu_3_Se_2_ clusters J Phys Chem C 2007 111 6658 6662

[B15] Hu JQ Meng XM Jiang Y Lee CS Lee ST Fabrication of germanium-filled silica nanotubes and aligned silica nanofibers Adv Mater 2003 15 70 73 10.1002/adma.200390014

[B16] Wu GS Zhang LD Cheng BC Xie T Yuan XY Synthesis of Eu_2_O_3_ nanotube arrays through a facile sol–gel template approach J Am Chem Soc 2004 126 5976 5977 10.1021/ja039012l 15137757

[B17] Mu C Yu YX Wang RM Wu K Xu DS Guo GL Uniform metal nanotube arrays by multistep template replication and electrodeposition Adv Mater 2004 16 1550 1553 10.1002/adma.200400129

[B18] Lee W Yoo H-I Lee J-K Template route toward a novel nanostructured superionic conductor film; AgI nanorod/γ-Al_2_O_3_ Chem Commun 2001 2530 2531

[B19] Xu NS Huq SE Novel cold cathode materials and applications Mater Sci Eng R 2005 48 47 189 10.1016/j.mser.2004.12.001

[B20] Du XS Yu ZZ Dasari A Ma J Meng YZ Mai YW Facile synthesis and assembly of Cu_2_S nanodisks to corncoblike nanostructures Chem Mater 2006 18 5156 5158 10.1021/cm0617135

[B21] Feng XP Li YX Liu HB Li YL Cui S Wang N Jiang L Liu XF Yuan MJ Controlled growth and field emission properties of CuS nanowalls Nanotechnology 2007 18 145706 10.1088/0957-4484/18/14/145706

[B22] Sakamoto T Sunamura H Kawaura H Hasegawa T Nakayama T Aono M Nanometer-scale switches using copper sulfide Appl Phys Lett 2003 82 3032 3034 10.1063/1.1572964

[B23] Sagade A Copper sulphide (Cu_x_S) as an ammonia gas sensor working at room temperature Sens Actuators B 2008 133 135 143 10.1016/j.snb.2008.02.015

[B24] Neville RC Solar Energy Conversion: the Solar Cell 1995 2 Amsterdam: Elsevier

[B25] Hodes G Manassen J Cahen D Electrocatalytic electrodes for the polysulfide redox system J Electrochem Soc 1980 127 544 549 10.1149/1.2129709

[B26] Liu ZP Xu D Liang JB Shen JM Zhang SY Qian YT Growth of Cu_2_S ultrathin nanowires in a binary surfactant solvent J Phys Chem B 2005 109 10699 10704 10.1021/jp050332w 16852299

[B27] Larsen TH Sigman M Ghezelbash A Christopher Doty R Korgel BA Solventless synthesis of copper sulfide nanorods by thermolysis of a single source thiolate-derived precursor J Am Chem Soc 2003 125 5638 5639 10.1021/ja0342087 12733895

[B28] Mehdi MK Masoud SN Majid R Preparation and characterization of Cu_2_S nanoparticles via ultrasonic method J Clust Sci 2013 24 927 934 10.1007/s10876-012-0537-0

[B29] Chen YB Chen L Wu LM The structure-controlling solventless synthesis and optical properties of uniform Cu_2_S nanodisk Chem Eur J 2008 14 11069 11075 10.1002/chem.200801447 19003830

[B30] Wu Y Wadia C Ma WL Sadtler B Paul Alivisatos A Synthesis and photovoltaic application of copper(I) sulfide nanocrystals Nano Lett 2008 8 2551 2555 10.1021/nl801817d 18651779

[B31] Liu X Wang XL Zhou B Law WC Cartwright AN Swihart MT Size-controlled synthesis of Cu_2-*x*_E (E = S, Se) nanocrystals with strong tunable near-infrared localized surface plasmon resonance and high conductivity in thin films Adv Funct Mater 2013 23 1256 1264 10.1002/adfm.201202061

[B32] Zhang HT Wu G Chen XH Large-scale synthesis and self-assembly of monodisperse hexagon Cu_2_S nanoplates Langmuir 2005 21 4281 4282 10.1021/la050741j 16032836

[B33] Zhu YF Fan DH Shen WZ A general chemical conversion route to synthesize various ZnO-based core/shell structures J Phys Chem C 2008 112 10402 10406 10.1021/jp802545e

[B34] Zhu YF Fan DH Shen WZ Chemical conversion synthesis and optical properties of metal sulfide hollow microspheres Langmuir 2008 24 11131 11136 10.1021/la801523h 18720954

[B35] Shuai XM Shen WZ A facile chemical conversion synthesis of ZnO/ZnS core/shell nanorods and diverse metal sulfide nanotubes J Phys Chem C 2011 115 6415 6422 10.1021/jp2005716

[B36] Shuai XM Shen WZ A facile chemical conversion synthesis of Sb_2_S_3_ nanotubes and the visible light-driven photocatalytic activities Nanoscale Res Lett 2012 7 199 10.1186/1556-276X-7-199 22448960 PMC3331828

[B37] Deng MH Zhang QX Huang SQ Li DM Luo YH Shen Q Toyoda T Meng QB Low-cost flexible nano-sulfide/carbon composite counter electrode for quantum-dot-sensitized solar cell Nanoscale Res Lett 2010 5 986 990 10.1007/s11671-010-9592-3 20672135 PMC2893919

[B38] Dloczik L Konenkamp R Nanostructure transfer in semiconductors by ion exchange Nano lett 2003 3 651 653 10.1021/nl0340879

[B39] Kumar B Gong H Chow SY Tripathy S Hua YN Photoluminescence and multiphonon resonant Raman scattering in low-temperature grown ZnO nanostructures Appl Phys Lett 2006 89 071922 10.1063/1.2336997

[B40] Weast RC CRC Handbook of Chemistry and Physics 1988 69 Boca Raton: CRC Press

[B41] Minceva-Sukarova B Najdoski M Grozdanov I Chunnilall CJ Raman spectra of thin solid films of some metal sulfides J Mol Struct 1997 410–411 267 270

[B42] Wang SY Wang W Lu ZH Asynchronous-pulse ultrasonic spray pyrolysis deposition of Cu_x_S (x = 1, 2) thin films Mater Sci Eng B 2003 103 184 188 10.1016/S0921-5107(03)00199-5

[B43] Lai CX Wu QB Chen J Wen LS Ren S Large-area aligned branched Cu_2_S nanostructure arrays: room-temperature synthesis and growth mechanism Nanotechnology 2010 21 215602 10.1088/0957-4484/21/21/215602 20431195

